# A Cross-Sectional Study Comparing Patient Information Guides for Amyotrophic Lateral Sclerosis, Myasthenia Gravis, and Guillain-Barré Syndrome Produced by ChatGPT-4 and Google Gemini 1.5

**DOI:** 10.7759/cureus.79646

**Published:** 2025-02-25

**Authors:** Daaniya Tariq, Ramya Madhusudan, Yashaswi Guntupalli, Shivaashish Karumanchi Anantha Venkata Sai, Bharath Vejandla, Mohit LNU

**Affiliations:** 1 Internal Medecine, Shaheed Suhrawardy Medical College and Hospital, Dhaka, BGD; 2 Internal Medicine, Karpaga Vinayaga Institute of Medical Science and Research Centre, Maduranthagam, IND; 3 Internal Medicine, Sri Venkateswara Institute of Medical Sciences (SVIMS), Tirupati, IND; 4 Internal Medicine, Tagore Medical College and Hospital, Chennai, IND; 5 Internal Medicine, All American Institute of Medical Science, Black River, JAM; 6 Internal Medicine, Rajendra Institute of Medical Sciences, Jharkhand, IND

**Keywords:** amyotrophic lateral sclerosis, artificial intelligence, chatgpt, google gemini, guillain-barré syndrome, myasthenia gravis

## Abstract

Introduction: Patient education for amyotrophic lateral sclerosis (ALS), myasthenia gravis (MG), and Guillain-Barré syndrome (GBS) is essential for effective symptom management, improving quality of life, and enabling informed care decisions. AI tools enhance healthcare and patient education through personalized care and improved diagnostics.

Methods: In this study, ChatGPT (OpenAI, San Francisco, CA, USA) and Google Gemini (Mountain View, CA, USA) generated patient education guides for ALS, MG, and GBS. Variables included word count, sentence count, average words and syllables per sentence, grade level, ease score using the Flesch-Kincaid calculator, similarity score using QuillBot, and reliability using a modified DISCERN score. Statistical analysis was done using R version 4.3.2 (2023; R Foundation for Statistical Computing, Vienna, Austria).

Results: ChatGPT-generated brochures for patient education on ALS, MG, and GBS had a higher grade level and lower ease score compared to those generated by Google Gemini. Although both models had similar reliability and similarity percentages, ChatGPT produced more content with greater complexity and slightly higher reliability.

Conclusion: This study found no significant difference in the average ease, grade, and reliability scores between the two AI tools when generating patient information brochures on ALS, MG and GBS. However, a statistically significant difference was observed in the mean word counts generated by the tools.

## Introduction

Neuro-muscular disorders such as amyotrophic lateral sclerosis (ALS), myasthenia gravis (MG), and Guillain-Barré syndrome (GBS) are severe conditions characterized by progressive muscle weakness and can lead to significant disability and mortality [1-3]. Effective patient education plays a crucial role in managing these disorders by aiding in the early identification of symptoms, proper medication management, and adherence to treatment plans, thereby reducing complications and improving patient outcomes. By empowering patients with knowledge about their condition, education becomes a pivotal tool in preventing early mortality and enhancing the quality of life [1,2].

In recent years, artificial intelligence (AI) tools like ChatGPT (OpenAI, San Francisco, CA, USA) and Google Gemini (Mountain View, CA, USA) have emerged as promising aids in patient education. These tools provide easily accessible, accurate information about a wide range of diseases, including complex neuromuscular disorders. ChatGPT, a conversational AI developed by OpenAI, is designed to simulate human-like dialogue, and can provide personalized educational content [4]. Google Gemini, another advanced AI tool, offers extensive health information, including up-to-date research findings and patient education materials [5]. While these AI tools enhance patient education, there are concerns regarding the accuracy of information, the lack of a personal connection with healthcare providers, and the potential for over-reliance on these technologies [4,5].

Combining the capabilities of ChatGPT and Google Gemini can significantly enhance patient counseling for neuromuscular disorders like ALS, MG, and GBS. These AI tools can provide patients with tailored, accessible information, complementing the guidance of healthcare professionals. However, careful consideration must be given to ensuring the accuracy of the information provided and integrating these tools into a holistic patient care approach [4-6].

Aims and objectives

This study aims to compare the readability, reliability, and content complexity of patient education materials generated by ChatGPT and Google Gemini for ALS, MG, and GBS.

## Materials and methods

This original cross-sectional study was carried out over the course of a week, beginning from the 5th of August 2024 to the 12th of August 2024. Ethics committee approval was deemed exempt in view of no human participants.

Three prevalent neurological conditions (ALS, MG, and GBS) were chosen. Google Gemini (Gemini 1.5 Flash) and ChatGPT (GPT-4 version) were the two AI tools used for generation of brochures for patient education [5,7]. Prompts were given to both AI tools: “Write a patient education guide for Amyotrophic Lateral Sclerosis (ALS)”; “Write a patient education guide for Myasthenia Gravis”; “Write a patient education guide for “Guillain-Barré Syndrome” (Appendices 1-3).

The responses generated by the AI were scored using a variety of statistical metrics and saved in a Microsoft (Redmond, WA, USA) Word document. Flesch-Kincaid Calculator was used to calculate measures like word count, number of sentences, average number of words per sentence, average number of syllables per word, grade level and ease score [8]. Quillbot plagiarism checker was used to measure the similarity percentage and modified DISCERN score was used to calculate the reliability score [9,10].

The modified DISCERN score is a set of five questions derived from the original DISCERN instrument for assessing the reliability of health information. Each question on the modified DISCERN scale was assigned a score of 0 or 1. A total score of 5 denotes great dependability, whereas a 0 indicates low reliability in this rating system.

The data was exported into Microsoft Excel for further analysis. The statistical analysis was done using R (version 4.3.2, R Foundation for Statistical Computing, Vienna, Austria), a programming language extensively used for statistical computing [6]. The responses generated by the AI tools were compared using unpaired T-test for which the P-value of <0.05 was considered significant. The linear correlation of ease score and reliability score was measured using Pearson’s correlation coefficient which has a value ranging from -1 to 1.

## Results

Table 1 compares the characteristics of responses generated by ChatGPT and Google Gemini across various metrics. ChatGPT responses are significantly longer (608 words on average) compared to Google Gemini (403 words), with a notable p-value of 0.0033. The responses also differ in average words per sentence and syllables per word, though these differences are not statistically significant. ChatGPT's responses tend to have a higher grade level (12.13) and lower ease score (26.90) compared to Google Gemini. Both models show similarities in response reliability and similarity percentage, with no statistically significant differences across these metrics.

**Table 1:** Characteristics of responses generated by ChatGPT and Google Gemini *t-test. P-values <0.05 in t-test are considered statistically significant.

Variables	ChatGPT		Google Gemini		P value
	Mean	Standard Deviation	Mean	Standard Deviation	
Words	608.0	42.04	403.0	32.05	0.0033*
Sentences	59.0	9.64	50.0	8.19	0.2869
Average Words per Sentence	10.57	2.25	8.17	0.76	0.1982
Average Syllables per Word	2.0	0.00	1.90	0.17	0.4226
Grade Level	12.13	0.86	10.0	2.10	0.2137
Ease Score	26.90	2.29	37.80	14.83	0.3303
Similarity %	35.20	4.31	33.57	18.10	0.8918
Reliability Score	4.0	1.00	3.67	1.15	0.7250

There was no significant difference in the sentence count (p=0.2869), average word per sentence (p=0.1982), average syllables per word (p=0.4226), grade level (p=0.2137), ease score (p=0.3303), similarity% (p=0.8918) and reliability score (p=0.7250) between ChatGPT and Google Gemini. However, the word count was significantly more for ChatGPT-generated responses compared to Google Gemini (p=0.0033).

**Figure 1:**
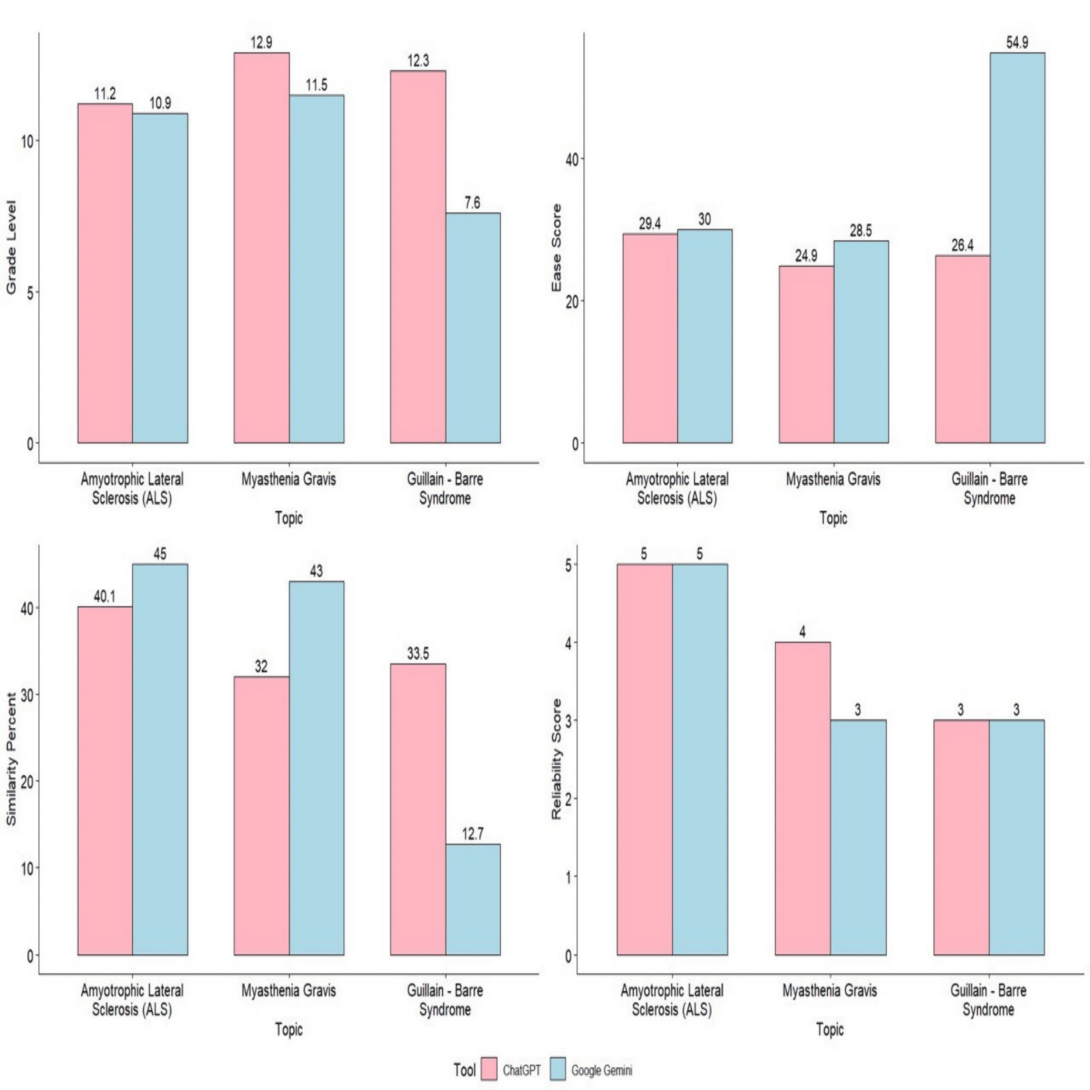
Graphical representation of comparison between grade level, ease score, similarity percent, and reliability score for the patient education guides generated by ChatGPT and Google Gemini

Figure 1 shows the grade level, ease score, similarity percent, and reliability score of the patient education guides made by ChatGPT and Google Gemini for the diseases ALS, MG, and GBS.

Based on Figure 1, the grade level for ChatGPT (ALS: 11.2, MG: 12.9, GBS: 12.3; mean = 11.9) was higher than Google Gemini (ALS: 10.9, MG: 11.5, GBS: 7.6; mean = 10). The ease score for Google Gemini (ALS: 30, MG: 28.5, GBS: 54.9; mean = 37.8) was higher than ChatGPT (ALS: 29.4, MG: 24.9, GBS: 26.4; mean = 26.9). The similarity percent for ChatGPT (ALS: 40.1, MG: 32, GBS: 33.5; mean = 35.2) was higher than Google Gemini (ALS: 45, MG: 43, GBS: 12.7; mean = 33.5). The reliability score was similar but slightly higher for ChatGPT (ALS: 5, MG: 4, GBS: 3; mean = 4) than Google Gemini (ALS: 5, MG: 3, GBS: 3; mean = 3.6). ChatGPT was slightly more complex than Google Gemini, but it was inferred to be more reliable. 

## Discussion

The responses produced by two AI systems, ChatGPT and Google Gemini, for patient education brochures for ALS, MG, and GBS were compared in a cross-sectional research. The analysis revealed that a statistically significant difference between ChatGPT and Google Gemini was in the number of words generated (p=0.0033). ChatGPT generates longer and slightly more complex text, while Google Gemini’s text may be easier to read. The tools were relatively similar in terms of reliability and the other assessed variables.

AI plays a pivotal role in transforming patient education by delivering customized and accessible information, aiding patients in making informed health decisions [11]. With AI’s rapid advancements and increasing integration in healthcare and educational contexts, the demand for succinct and comprehensible educational materials has grown [12]. In this analysis, ChatGPT generated an average of 608 words per text, and Google Gemini produced 403 words per text, a notable difference. Additionally, the average words per sentence were 10.57 for ChatGPT and 8.17 for Google Gemini. Although the National Institutes of Health (NIH) and the National Academy of Medicine (NAM) recommend that patient educational materials be written at or below a sixth-grade reading level, the Flesch-Kincaid scores indicated that ChatGPT’s content was at a 12th-grade level, while Google Gemini’s was at a 10th-grade level [8,13]. This reveals that both AI tools produced content that exceeds the readability of high school levels, with ChatGPT being particularly complex and Google Gemini requiring at least a 10th-grade reading level. A cross-sectional study by Gibson et al. further corroborates this finding, noting that AI-generated content for prostate cancer education was “difficult to read,” with a Flesch reading ease score of 45.97 and a Flesch-Kincaid grade level of 12.12 [14].

AI tools, including ChatGPT and Google Gemini, are extensively trained on pre-existing literature, and may sometimes generate content resembling previously published research, raising concerns about potential plagiarism. Additionally, these tools lack the ability to fully grasp the context of the literature, resulting in suggestions that are not always relevant, or sometimes biased [15]. When integrating AI into medical practice and research, it is crucial to avoid plagiarism, as it undermines the academic integrity of medical professionals and their credentials. The QuillBot plagiarism tool [9], used to assess the similarity percent, showed that ChatGPT had an average similarity score of 35.2% and Google Gemini had an average similarity score of 33.57%. The results highlight the increasing focus on ChatGPT’s applications in healthcare, underscoring the need for further investigation into its effectiveness and the associated ethical considerations [16]. 

The modified DISCERN score is a tool used to evaluate the reliability of health web pages based on specific criteria; it was utilized in the analysis to assess the reliability of ChatGPT and Google Gemini [17]. The average DISCERN score for ChatGPT was 4.0, while Google Gemini was 3.67. A cross-sectional study by Golan et al. revealed significant discrepancies between ChatGPT’s quality assessments and those of human evaluators, as well as between ChatGPT and established tools like Readable.com, indicating potential misalignment in evaluating the quality and readability of medical content [18].

Limitations

The study was limited to two AI tools, ChatGPT and Google Gemini, suggesting a need for broader assessment. The focus on a limited number of diseases calls for further research into other diseases. The version of ChatGPT used is outdated, potentially affecting the accuracy of its content. The rapid evolution of medical science raises concerns about the ability of these tools to provide the most up-to-date information.

## Conclusions

This study shows that, for patient information brochures on ALS, MG, and GBS, there is no discernible difference in the average ease score, grade score, and reliability score of responses produced by the two AI tools. The two AI tools' "words" differ from one another in a statistically significant way.

Further research should be conducted to investigate more AI technologies in diverse and contemporary disorders. Furthermore, it should be determined whether these technologies can create material in accordance with the latest updated guidelines. Tools should be upgraded to offer up-to-date and reliable information that can also be utilized by the general population.

## Appendices

**Table 2:** Responses generated by the AI-models for the prompt; "Write a patient education guide for “Amyotrophic Lateral Sclerosis (ALS)”

Write a patient education guide for “Amyotrophic Lateral Sclerosis (ALS)”		
AI	ChatGPT	Google Gemini
Response	Patient Education Guide: Amyotrophic Lateral Sclerosis (ALS) Understanding ALS What is ALS? Amyotrophic Lateral Sclerosis (ALS), also known as Lou Gehrig’s disease, is a progressive neurodegenerative disorder that affects nerve cells in the brain and spinal cord. This disease leads to the gradual degeneration and death of motor neurons, which are responsible for controlling voluntary muscles. Symptoms of ALS: • Early Symptoms: • Muscle weakness, especially in the arms and legs • Muscle cramps and twitches • Difficulty speaking or swallowing • Stiffness or spasticity • Progressive Symptoms: • Severe muscle weakness and atrophy • Difficulty with fine motor tasks (e.g., buttoning a shirt) • Trouble walking or maintaining balance • Respiratory difficulties due to weakened diaphragm muscles Causes and Risk Factors: • Genetics: 5-10% of cases are familial, meaning they are inherited. • Environmental Factors: Possible links include exposure to toxins, heavy metals, or repetitive physical trauma, though exact causes are still not well understood. • Age and Gender: ALS is most common in people between the ages of 40 and 70, and it is slightly more common in men than in women. Diagnosis How is ALS Diagnosed? • Clinical Examination: A thorough neurological exam by a specialist to assess muscle strength, reflexes, and coordination. • Electromyography (EMG): Measures the electrical activity of muscles. • Nerve Conduction Studies (NCS): Tests the speed and strength of signals traveling in the nerves. • MRI: Helps rule out other conditions with similar symptoms. • Blood and Urine Tests: To exclude other diseases. • Genetic Testing: Especially if there is a family history of ALS. Treatment and Management Is there a Cure for ALS? Currently, there is no cure for ALS. However, there are treatments and therapies that can help manage symptoms and improve quality of life. Medications: • Riluzole (Rilutek): May slow disease progression. • Edaravone (Radicava): Can slow the decline in daily functioning. Therapies: • Physical Therapy: Helps maintain muscle strength and mobility. • Occupational Therapy: Assists in adapting daily activities to maintain independence. • Speech Therapy: Aids in addressing speech and swallowing difficulties. • Nutritional Support: Ensures adequate nutrition, which can become challenging as swallowing difficulties progress. • Respiratory Care: Non-invasive ventilation or other respiratory support to assist with breathing. Supportive Care: • Multidisciplinary Care Teams: Coordinated care involving neurologists, therapists, nutritionists, and other healthcare professionals. • Palliative Care: Focuses on comfort and quality of life, managing pain, and addressing emotional and spiritual needs. Living with ALS Daily Living Adjustments: • Home Modifications: Install ramps, grab bars, and stair lifts to improve accessibility. • Assistive Devices: Use wheelchairs, walkers, communication devices, and adapted utensils to aid in daily activities. Emotional and Psychological Support: • Counseling: Individual or family counseling can help manage the emotional impact of the disease. • Support Groups: Connecting with others who have ALS can provide emotional support and practical advice. Planning for the Future: • Advance Directives: Discuss and document your wishes regarding medical care and end-of-life decisions. • Legal and Financial Planning: Address wills, power of attorney, and financial planning to ensure your wishes are honored and your family is supported. Resources Organizations and Support: • ALS Association: Offers resources, support, and advocacy. • Muscular Dystrophy Association (MDA): Provides services and support for individuals with ALS. • Local Support Groups: Many communities have support groups for people with ALS and their families. Online Resources: • ALS Association Website: www.als.org • MDA Website: www.mda.org Remember: ALS affects everyone differently. Working closely with your healthcare team to create a personalized care plan is crucial in managing the disease and maintaining the best possible quality of life.	Understanding Amyotrophic Lateral Sclerosis (ALS) What is ALS? Amyotrophic Lateral Sclerosis (ALS), often referred to as Lou Gehrig's disease, is a progressive neurodegenerative disease that affects nerve cells in the brain and spinal cord. These nerve cells control the muscles that allow us to move, speak, swallow, and breathe. As the disease progresses, it causes weakness and muscle wasting, leading to increasing difficulty with these functions. Symptoms of ALS Symptoms of ALS can vary from person to person and may include: Muscle weakness and twitching: Often starting in the hands, feet, or limbs. Difficulty speaking, swallowing, and chewing. Breathing problems. Slurred speech. Muscle cramps and stiffness. Weight loss. It's important to note that not everyone with ALS experiences all of these symptoms. How is ALS Diagnosed? Diagnosing ALS can be challenging as the symptoms can mimic other conditions. Your doctor will conduct a thorough physical exam, review your medical history, and may order tests such as: Electromyography (EMG): Measures muscle electrical activity. Nerve conduction study: Evaluates nerve function. MRI: To rule out other conditions. Living with ALS There is currently no cure for ALS, but treatments can help manage symptoms and improve quality of life. Managing Symptoms: Physical therapy to maintain muscle strength and flexibility. Occupational therapy to assist with daily living activities. Speech therapy to improve communication. Respiratory therapy to manage breathing difficulties. Nutrition to maintain a healthy weight. Medications to slow disease progression or manage specific symptoms. Emotional Support: Support groups, counseling, and talking to loved ones can help you cope with the emotional challenges of ALS. Advance Care Planning: It's important to discuss your end-of-life wishes with your family and healthcare providers. Research and Hope While there is no cure, ongoing research is focused on understanding the causes of ALS and developing new treatments. Participating in clinical trials can contribute to finding a cure. Day-to-Day Living with ALS Energy Conservation: Pace yourself and prioritize activities. Communication: Explore assistive devices like speech-generating devices or communication boards. Independence: Maintain as much independence as possible with assistive technology and support. Caregiver Support: Involve family and caregivers in your care plan. Resources There are several organizations dedicated to providing support and information about ALS. Some of these include: The ALS Association Muscular Dystrophy Association (MDA) Amyotrophic Lateral Sclerosis Society (ALSA) Remember, you are not alone. Connect with other people living with ALS through support groups and online communities. This guide is intended to provide general information and should not replace medical advice. Always consult with your healthcare provider for personalized guidance. Would you like to learn more about a specific aspect of ALS, such as symptom management, caregiving, or available treatments?

**Table 3:** Responses for the prompt: "Write a patient education guide for “Myasthenia Gravis”"

Write a patient education guide for “Myasthenia Gravis”		
AI	ChatGPT	Google Gemini
Response	Myasthenia Gravis: A Patient Education Guide Introduction Myasthenia Gravis (MG) is a chronic autoimmune neuromuscular disorder characterized by weakness and rapid fatigue of voluntary muscles. It affects the communication between nerves and muscles, leading to muscle weakness. While MG is a manageable condition, understanding the disease and its treatment options is crucial for living a healthy life. What Causes Myasthenia Gravis? MG is caused by a breakdown in the communication between nerves and muscles. Normally, nerve cells release a chemical called acetylcholine, which binds to receptors on muscles to trigger contraction. In MG, the body’s immune system mistakenly attacks these receptors, preventing the muscles from contracting properly. Symptoms of Myasthenia Gravis The symptoms of MG can vary widely and may include: - Muscle Weakness: This is the most common symptom and typically worsens with activity and improves with rest. - Drooping Eyelids (Ptosis): Weakness in the muscles controlling the eyelids can cause one or both eyelids to droop. - Double Vision (Diplopia): Weakness in the muscles that control eye movement can lead to double vision. - Difficulty Swallowing (Dysphagia): The muscles involved in swallowing may be affected, leading to choking or difficulty swallowing. - Speech Difficulties: Weakness in the muscles controlling speech can result in slurred or soft speech. - Difficulty Breathing: In severe cases, the muscles involved in breathing can become weak, leading to respiratory issues. Diagnosing Myasthenia Gravis Diagnosis of MG typically involves several tests, including: - Physical and Neurological Examination: Your doctor will assess muscle strength, reflexes, and coordination. - Blood Tests: To detect antibodies that interfere with nerve-muscle communication. - Electromyography (EMG): This test measures the electrical activity of muscles to detect muscle weakness. - Edrophonium Test: A short-acting drug is given to temporarily improve muscle strength, indicating MG. - Imaging: A CT scan or MRI may be used to check for abnormalities in the thymus gland, often associated with MG. Treatment Options While there is no cure for MG, several treatment options can help manage symptoms and improve quality of life: - Medications: - Anticholinesterase Medications: Drugs like pyridostigmine help improve communication between nerves and muscles. - Immunosuppressants: Medications such as prednisone or azathioprine reduce the immune system's attack on the neuromuscular junction. - Monoclonal Antibodies: Drugs like rituximab or eculizumab target specific parts of the immune system to reduce symptoms. - Thymectomy: Removal of the thymus gland can reduce symptoms in some patients, especially if a thymoma (tumor of the thymus) is present. - Plasmapheresis and Intravenous Immunoglobulin (IVIG): These treatments remove or neutralize harmful antibodies in the blood, providing short-term relief, especially during a myasthenic crisis. Managing Myasthenia Gravis Living with MG requires careful management and lifestyle adjustments: - Regular Follow-Ups: Consistent monitoring by your healthcare provider is crucial for managing symptoms and adjusting treatment. - Healthy Lifestyle: Regular exercise, a balanced diet, and adequate rest can help manage symptoms. - Medication Adherence: Take medications as prescribed and communicate with your healthcare provider about any side effects. - Avoid Triggers: Infections, stress, and certain medications can worsen MG symptoms. Work with your healthcare provider to identify and avoid these triggers. - Emergency Plan: Have a plan in place in case of a myasthenic crisis, which involves severe muscle weakness leading to respiratory failure. This is a medical emergency. Support and Resources Living with MG can be challenging, but support is available: - Support Groups: Connecting with others who have MG can provide emotional support and practical advice. - Patient Education: Learn as much as you can about MG. Knowledge empowers you to make informed decisions about your care. - Healthcare Team: Your neurologist, primary care provider, and other specialists are there to support you. Don’t hesitate to ask questions or express concerns. Conclusion Myasthenia Gravis is a manageable condition with the right treatment and lifestyle adjustments. By understanding the disease, staying informed about treatment options, and working closely with your healthcare team, you can lead a fulfilling life with MG. If you have any further questions or concerns, don't hesitate to reach out to your healthcare provider.	Understanding Myasthenia Gravis What is Myasthenia Gravis? Myasthenia gravis (MG) is a chronic autoimmune disorder that causes muscle weakness. It happens when the body's immune system mistakenly attacks the neuromuscular junction, the place where nerves meet muscles. This interference disrupts the communication between nerves and muscles, leading to weakness. Symptoms of Myasthenia Gravis Symptoms can vary from person to person but often include: Muscle weakness: This is the primary symptom, often worsening with activity and improving with rest. Eye problems: Drooping eyelids (ptosis) and double vision (diplopia) are common. Difficulty swallowing (dysphagia): This can make eating and drinking challenging. Difficulty speaking (dysarthria): Speech may become slurred or weak. Breathing problems: In severe cases, muscle weakness can affect the muscles involved in breathing. Causes of Myasthenia Gravis The exact cause of MG is unknown, but it's believed to be an autoimmune disorder. The immune system mistakenly produces antibodies that attack the acetylcholine receptors at the neuromuscular junction. Diagnosis of Myasthenia Gravis Your doctor may diagnose MG based on your medical history, physical examination, and tests like: Blood tests to check for antibodies Electromyography (EMG) to measure muscle electrical activity Repetitive nerve stimulation test Imaging tests (CT or MRI) to check for thymus gland abnormalities Treatment of Myasthenia Gravis There's no cure for MG, but treatments can help manage symptoms and improve quality of life. These may include: Medications: To improve muscle strength and reduce symptoms. Immunotherapy: To suppress the immune system's attack on the neuromuscular junction. Plasmapheresis: To remove harmful antibodies from the blood. Thymectomy: Surgical removal of the thymus gland, which can be involved in MG. Living with Myasthenia Gravis Managing MG requires a balance of medical treatment, lifestyle adjustments, and support. Here are some tips: Energy conservation: Plan activities to avoid fatigue. Avoid triggers: Identify factors that worsen symptoms and try to minimize exposure. Regular exercise: Gentle exercises can help maintain muscle strength. Support groups: Connect with others living with MG for emotional support and information sharing. Emergency plan: Develop a plan for managing crises, such as a myasthenic crisis (severe muscle weakness affecting breathing). When to Seek Emergency Care Contact your doctor immediately if you experience: Difficulty breathing Swallowing problems Changes in speech or voice Sudden weakness Remember, while Myasthenia Gravis can be challenging, with proper management, you can lead a fulfilling life.

**Table 4:** Responses for the prompt: "Write a patient education guide for “Guillain – Barre Syndrome”"

Write a patient education guide for “Guillain – Barre Syndrome”		
AI	ChatGPT	Google Gemini
Response	Guillain-Barré Syndrome: A Patient Education Guide Introduction Guillain-Barré Syndrome (GBS) is a rare neurological disorder in which the body's immune system mistakenly attacks part of the peripheral nervous system. This condition can lead to muscle weakness, numbness, and, in severe cases, paralysis. While the exact cause of GBS is unknown, it often follows a respiratory or gastrointestinal infection. Most people recover from GBS, but recovery can take weeks, months, or even years. What Causes Guillain-Barré Syndrome? GBS occurs when the immune system attacks the myelin sheath, a protective layer around the nerves in the peripheral nervous system. This damage disrupts the transmission of signals between the nerves and muscles, leading to symptoms. The exact trigger for this immune response is unclear, but GBS often follows infections, surgeries, or vaccinations. The most commonly associated infections include: - **Respiratory Infections:** Such as the flu or other viral illnesses. - **Gastrointestinal Infections:** Especially those caused by the bacterium Campylobacter jejuni. - **Other Infections:** Such as Zika virus, Epstein-Barr virus, and cytomegalovirus. Symptoms The symptoms of GBS usually start with weakness and tingling in the legs, which can spread to the upper body. Common symptoms include: - **Muscle Weakness:** Starting in the legs and progressing to the arms and upper body. - **Tingling Sensations:** Often starting in the toes and fingers. - **Loss of Reflexes:** Reduced or absent reflexes in the affected limbs. - **Difficulty Walking:** Due to muscle weakness or unsteady gait. - **Difficulty Breathing:** If the muscles involved in breathing are affected. - **Pain:** Aching or cramp-like pain, which may worsen at night. Diagnosis Diagnosing GBS involves a combination of clinical evaluation and diagnostic tests, including: - **Neurological Examination:** To assess muscle strength, reflexes, and sensation. - **Lumbar Puncture (Spinal Tap):** To check for elevated protein levels in cerebrospinal fluid, a common finding in GBS. - **Electromyography (EMG) and Nerve Conduction Studies (NCS):** To assess the electrical activity in the muscles and the speed of nerve signals. Treatment There is no cure for GBS, but several treatments can help manage symptoms and speed recovery: - **Intravenous Immunoglobulin (IVIG):** A treatment that provides the body with healthy antibodies to help neutralize harmful antibodies. - **Plasmapheresis (Plasma Exchange):** A procedure that removes harmful antibodies from the blood. - **Supportive Care:** Including physical therapy, pain management, and respiratory support if needed. Recovery and Prognosis Recovery from GBS can vary from person to person. While most people recover fully, some may experience lingering symptoms such as weakness, numbness, or fatigue. The recovery process can take weeks to several years, and early treatment is crucial for better outcomes. About 15-20% of people may have permanent residual weakness, and in rare cases, GBS can be life-threatening due to complications like respiratory failure. Living with Guillain-Barré Syndrome Managing life with GBS involves physical rehabilitation, emotional support, and adjusting to temporary or long-term changes in mobility and function. Here are some tips: - **Rehabilitation:** Physical and occupational therapy can help regain strength, coordination, and independence. - **Support Systems:** Family, friends, and support groups can provide emotional and practical support. - **Healthy Lifestyle:** Maintain a balanced diet, avoid infections, and practice good hygiene to support overall health. - **Monitoring:** Regular follow-up with healthcare providers to monitor progress and manage any complications. When to Seek Medical Help Seek immediate medical attention if you experience: - Difficulty breathing or shortness of breath. - Severe muscle weakness or paralysis. - Difficulty swallowing or speaking. Conclusion Guillain-Barré Syndrome is a serious condition that requires prompt medical attention and supportive care. While recovery can be challenging, many people recover fully or significantly. Working closely with healthcare providers and having a strong support system can make a big difference in managing and recovering from GBS. For more information or support, contact your healthcare provider or local GBS support group.	Guillain-Barré Syndrome (GBS): Understanding Your Condition What is Guillain-Barré Syndrome (GBS)? Guillain-Barré Syndrome (GBS) is a rare autoimmune disorder where the body's immune system mistakenly attacks the nerves. This can lead to muscle weakness, numbness, and in severe cases, paralysis. While it sounds scary, most people recover fully from GBS. Symptoms of GBS Symptoms can vary from person to person but often include: Weakness that starts in the feet and legs and spreads upward Numbness or tingling in the hands and feet Difficulty walking or climbing stairs Pain in the muscles or joints Difficulty with facial muscles (e.g., smiling, chewing, closing eyes) Shortness of breath Difficulty swallowing Changes in heart rate or blood pressure Important: If you experience any of these symptoms, seek medical attention immediately. Causes of GBS The exact cause of GBS is unknown, but it often occurs after an infection (like a cold or flu). In some cases, it can be triggered by vaccinations or surgery. Treatment for GBS Treatment focuses on managing symptoms and helping the body recover. It may include: Plasma exchange: Removing harmful antibodies from the blood. Immunoglobulin therapy: Injecting healthy antibodies to fight the disease. Supportive care: This includes managing pain, preventing blood clots, and helping with breathing if necessary. Physical therapy: To help regain strength and mobility. Occupational therapy: To help with daily living activities. Recovery from GBS Recovery from GBS is a gradual process. While some people recover fully within a few weeks, others may take months or even years. Regular physical and occupational therapy are key to a successful recovery. Living with GBS GBS can be a challenging experience, but with proper support and care, you can manage your condition and live a fulfilling life. Some tips include: Build a support system: Family, friends, and support groups can provide emotional and practical support. Manage fatigue: Rest when needed and avoid overexertion. Maintain a healthy lifestyle: Eat a balanced diet, get enough sleep, and manage stress. Regular check-ups: Keep appointments with your healthcare provider to monitor your condition. When to Seek Medical Attention Contact your doctor immediately if you experience: Worsening weakness Difficulty breathing Changes in heart rate or blood pressure Remember, you are not alone. Many people have successfully recovered from GBS. With the right treatment and support, you can overcome this challenge.
